# Supplementary material for: Resolving the full spectrum of human genome variation using Linked-Reads
Source: Genome Res. 2019 Apr;29(4):635–45. doi: 10.1101/gr.234443.118 (PMC6442396; doi:10.1101/gr.234443.118)
Supplement: Supplemental Material [file supp_gr.234443.118_Supplemental_longranger-2.2.zip › longranger-2.2.tar.bz2!/lr2.2-public/lib/stan/cmdstan/stan/doc/api/html/classstan_1_1model_1_1prob__grad-members.html]

Stan: Member List


|  |  |
| --- | --- |
|  | Stan  2.5.0  probability, sampling & optimization |


- Main Page
- Related Pages
- Namespaces
- Classes
- Files

- Class List
- Class Index
- Class Hierarchy
- Class Members

All Classes Namespaces Files Functions Variables Typedefs Enumerations Enumerator Friends Macros Pages

- stan
- model
- prob\_grad

stan::model::prob\_grad Member List

This is the complete list of members for stan::model::prob\_grad, including all inherited members.

|  |  |  |
| --- | --- | --- |
| num\_params\_i() const | stan::model::prob\_grad | inline |
| num\_params\_r() const | stan::model::prob\_grad | inline |
| num\_params\_r\_\_ | stan::model::prob\_grad | protected |
| param\_range\_i(size\_t idx) const | stan::model::prob\_grad | inline |
| param\_ranges\_i\_\_ | stan::model::prob\_grad | protected |
| prob\_grad(size\_t num\_params\_r) | stan::model::prob\_grad | inline |
| prob\_grad(size\_t num\_params\_r, std::vector< std::pair< int, int > > &param\_ranges\_i) | stan::model::prob\_grad | inline |
| ~prob\_grad() | stan::model::prob\_grad | inlinevirtual |


---

[ Stan Home Page ]

*© 2011–2014,
Stan Development Team.*
